# The use of a *Cissus quadrangularis *formulation in the management of weight loss and metabolic syndrome

**DOI:** 10.1186/1476-511X-5-24

**Published:** 2006-09-02

**Authors:** Julius Oben, Dieudonne Kuate, Gabriel Agbor, Claudia Momo, Xavio Talla

**Affiliations:** 1 Department of Biochemistry, University of Yaoundé I, Yaoundé, Cameroon; 2 Institute of Medical Research & Medicinal Plant studies, Yaounde, Cameroon

## Abstract

**Aim:**

Once considered a problem of developed countries, obesity and obesity-related complications (such as metabolic syndrome) are rapidly spreading around the globe. The purpose of the present study was to investigate the use of a *Cissus quadrangularis *formulation in the management of metabolic syndrome, particularly weight loss and central obesity.

**Methods:**

The study was a randomized, double-blind, placebo-controlled design involving 123 overweight and obese persons (47.2% male; 52.8% female; ages 19–50). The 92 obese (BMI >30) participants were randomized into three groups; placebo, formulation/no diet, and formulation/diet (2100–2200 calories/day). The 31 overweight participants (BMI = 25–29) formed a fourth (no diet) treatment group. All participants received two daily doses of the formulation or placebo and remained on a normal or calorie-controlled diet for 8 weeks.

**Results:**

At the end of the trial period, statistically significant net reductions in weight and central obesity, as well as in fasting blood glucose, total cholesterol, LDL-cholesterol, triglycerides, and C-reactive protein were observed in participants who received the formulation, regardless of diet.

**Conclusion:**

*Cissus quadrangularis *formulation appears to be useful in the management of weight loss and metabolic syndrome.

## Background

Although still defined in diverse terms, metabolic syndrome is a common disorder arising as a result of the increased prevalence of obesity throughout the world [1]. Metabolic syndrome, also known as insulin resistance syndrome and Syndrome X, has 3 main potential etiologic categories: obesity and disorders of adipose tissue; insulin resistance; and a constellation of independent factors (e.g., molecules of hepatic, vascular, and immunologic origin) that mediate specific components of the metabolic syndrome [2].

In the United States, over 60% of the adult population is now overweight or obese [3] and 47 million people have metabolic syndrome, which will soon overtake cigarette smoking as the number one risk factor for heart disease [4,5]. Globally, the disorder has become a major public health challenge. (In Cameroon, approximately 25% of the population is now considered obese.)

Obesity has been shown to contribute to high serum cholesterol, low HDL cholesterol and hyperglycemia, all of which increase the chances of cardiovascular disease (CVD) [6-8]. Correlations between central obesity and high blood pressure, high blood cholesterol, low levels of high density lipoprotein-cholesterol, and high fasting blood glucose levels have been shown for both sexes in various racial and ethnic groups [6-10].

Since it has been determined that abdominal fat poses a greater health risk than fat stored in the lower half of the body [11], waist circumference has become a major factor in body-fat assessment [12,13]. A large waist circumference (>88 cm in women and 102 cm in men) is associated with an increased risk for type 2 diabetes, dyslipidemia, hypertension and CVD in patients with a body mass index (BMI) 25–34 [8]. Moreover, for obese patients with metabolic complications, changes in waist circumference are useful predictors of CVD risk factors [13,14]. Thus, in addition to weight (kg), fat (%) and BMI, changes in waist measurement (cm) was one of the primary endpoints in this study.

Because of its links with obesity, it is difficult to identify a unique role for insulin resistance in patients with metabolic syndrome. Although insulin resistance generally rises with increasing body fat content, one finds a broad range of insulin sensitivities at all levels [6]. Although most people with BMI ≥ 30 have postprandial hyperinsulinemia and relatively low insulin sensitivity, there is variation in insulin sensitivity even within the obese population [6,7]. Overweight persons (BMI 25–29) also exhibit a spectrum of insulin sensitivities, which seems to suggest an inherited component to insulin resistance.

The increased chance of CVD and type 2 diabetes require therapeutic consideration for the vast numbers of overweight/obese persons now at high risk for these diseases [1]. The current International Diabetes Foundation recommendations for preventing or delaying the development of diabetes include both primary and secondary interventions. The former emphasizes lifestyle changes such as calorie restriction and increased physical activity, and the latter (for people at high risk for CVD) uses pharmacological agents [15] that specifically target individual components of metabolic syndrome [15-20]. When used by obese patients in combination with dietary regimes, these agents can produce some weight loss and some reversal of accompanying complications. The role of pharmacotherapy, however, has been compromised by safety issues leading to the withdrawal of some medications from the market [21,22]. The combination of safety concerns and high costs have forced many populations to continue to rely on traditional healing methods using the indigenous pharmacopoeia.

*Cissus quadrangularis*, for example, is used by common folk in India to hasten the fracture healing process [23-28]. In Cameroon, the whole plant is used in oral re-hydration, while in Africa and Asia the leaf, stem, and root extracts are utilized in the management of various ailments [29-33]. Phytochemical analyses of *Cissus quadrangularis *reveal a high content of ascorbic acid, carotene, phytosterol substances and calcium [34,35], and there have also been reports of the presence of β-sitosterol, δ-amyrin and δ-amyrone [36]. All these components have potentially different metabolic and physiologic effects [37,38]. Although researchers have investigated several uses of Cissus quadrangularis, its potential application against metabolic syndrome has not yet been reported.

Several other dietary supplements (green tea, soy, chromium, selenium, B-vitamins) have only marginal effects in treating obesity but they address other metabolic syndrome symptoms and thus were included in the formulation. Green tea (*Camellia sinensis*) extracts contain high concentrations of epigallocatchin gallate and may work with other chemicals to increase levels of fat oxidation and thermogenesis [39,40]. Numerous studies on soy (*Glycine max*) protein show it is associated with a reduction in serum cholesterol and triglyceride levels and suggest it may protect against the development of coronary heart disease [41]. Chromium helps insulin metabolize fat, turn protein into muscle, and convert sugar into energy [42]; thus, chromium supplementation can favorably influence glucose/insulin metabolism, reduce levels of harmful LDL cholesterol, and increase HDL cholesterol [43]. Both humans and animals require selenium for the optimal functioning of the *selenoproteins*, which reduce the risk of CVD by decreasing lipid peroxidation and influencing the metabolism of the cell-signaling prostaglandins [44]. Lastly, B-vitamins (B-6, B-12 and folic acid) regulate energy metabolism [45], which plays a critical role in obesity management; they also maintain lower homocysteine levels [46], which are closely associated with cardiovascular health benefits.

The purpose of the present study was to examine the efficacy of a *Cissus quadrangularis *formulation (Cylaris™) containing the above agents in the management of obesity and metabolic syndrome.

## Methods

The study was a prospective, randomized, double blind, placebo-controlled design conducted by the Laboratory for Nutrition and Nutritional Biochemistry at the University of Yaoundé I, Cameroon, Africa. The Cameroon National Ethics Committee approved the protocol. Applicants were advised of the study's purpose, nature, and potential risks, and all gave their written informed consent before participation. The study was conducted in accordance with the Helsinki Declaration (1983 version).

### Participants

Eligibility criteria included meeting the minimal standards for overweight (i.e., a BMI >25 and a waist circumference >85.5 cm.) and a willingness to participate in an 8-week trial. Exclusion criteria (confirmed via an initial interview and physical examination) included pregnancy/lactatation, use of any form of weight-reducing medication, participation in intense exercise programs, medical conditions known to affect serum lipids, and a history of drug or alcohol abuse. For the 123 eligible participants, BMIs ranged from 25.5 to 45.6; waist circumferences from 85.5 cm to 125 cm; and weight from 62.6 kg to 142 kg. (See Table 1 for other baseline characteristics.) Ninety-two persons qualified as obese (BMI >30), and 31 as overweight (BMI = 25–29). The age range was 19 to 50; males = 47.2%; females = 52.8%.

**Table 1:** Baseline (week 0) characteristics of study participants

BMI	Greater than 30 (Obese)			25 – 29 (Overweight)
Treatment	Placebo (n = 30)	Formulation/no diet (n = 31)	Formulation/Diet (n = 31)	Formulation/no diet (n = 31)

**Demographic Characteristics **(no.)				
Sex				
Male	14	15	14	15
Female	16	16	17	16
Age (years)*	39.9 (9.6)	41.0 (9.4)	40.9 (9.8)	39.7 (9.9)
Race or ethnic group				
West African	30	31	31	31
Family History of Diabetes	2	1	0	2
History of Gestational Diabetes	2	2	3	2
				
Anthropomorphic Characteristics*^1^				
Weight (kg)	95.6 (14.3)	95.8 (8.0)	95.3 (9.1)	76.3 (7.9)
Fat (%)	41.8 (5.2)	46.5 (5.8)	48.1 (3.0)	35.8 (5.2)
Body Mass Index (kg/m^2^)	38.4 (1.6)	37.7 (1.5)	37.5 (1.0)	27.3 (0.7)
Waist Circumference (cm)	103.4 (8.1)	98.6 (4.6)	97.8 (5.7)	86.9 (3.2)
				
Serological Characteristics*^2^				
Total Cholesterol (mg/dl)	160.8 (18.0)	159.1 (30.7)	171.0 (24.4)	152.6 (26.9)
LDL Cholesterol (mg/dl)	103.5 (14.9)	99.8 (11.7)	116.6 (15.4)	101.6 (17.0)
HDL Cholesterol (mg/dl)	32.7 (7.5)	36.6 (8.0)	38.6 (7.1)	44.4 (9.8)
Triglycerides (mg/dl)	142.5 (31.8)	156.0 (25.3)	144.9 (30.0)	117.4 (25.3)
C-Reactive Protein (mg/dl)	8.0 (1.1)	8.0 (1.0)	8.1 (0.7)	7.8 (0.8)
Fasting Blood Glucose (mg/dl)	101.6 (11.9)	101.2 (15.9)	102.4 (9.8)	93.3 (12.4)

* These data are shown as means with standard deviations in parentheses

^1 ^Primary outcome measures

^2 ^Secondary outcome measures

### Intervention

The 92 obese persons were randomized to a placebo or one of two treatment groups; the 31 overweight persons formed a fourth group. One obese treatment group was prescribed a calorie-controlled (2100–2200 calories/day) diet; none of the groups was prescribed an exercise regimen. Apart from the expected lower anthropomorphic and serological characteristics of the overweight group, none of the baseline differences between the groups was clinically significant.

The overweight group was used for general comparison with the obese groups, thus the results were expressed in percentages rather than absolute values. The formulation/diet group was used to determine if a short-term, calorie-controlled diet would significantly increase anthropomorphic and serological outcomes compared to the formulation/non-diet group.

### Materials

The *Cissus quadrangularis *formula, Cylaris™, contains a *Cissus quadrangularis *extract (supplied by Gateway Health Alliances, Inc, Fairfield, California, USA), standardized to contain a minimum of 2.5% phytosterols and a minimum of 15% soluble plant fiber. The formula also consists of a soy albumin extract (supplied by Gateway Health Alliances, Inc, Fairfield, California, USA); a green tea extract standardized to 22% EGCG and 40% caffeine; niacin bound chromium (ChromeMate™ supplied by InterHealth Nutraceuticals, Inc, Benicia, California, USA); selenium standardized to 0.5% l-Selenomethionine; vitamin B6 (as pyridoxine hydrochloride); vitamin B12 (as cyanocobalamin); and folic acid (supplied by Protein Research, Inc, Livermore, California, USA). All active and placebo capsules were manufactured and bottled by Protein Research, Inc.

Participants received two daily doses (514 mg each) of the *Cissus *formulation or placebo for 8 weeks. Each capsule was taken with 8–12 oz of water immediately prior to meals (preferably breakfast and dinner). In keeping with the experimental design, the capsules were identical in shape, color and appearance, and neither the participants nor researchers knew which capsule was administered. Side effects were noted on each visit.

Body weight and percentage of body fat were determined in the 12-hour- fasted participants with a Tanita™ BC-418 Segmental Body Composition Analyzer/Scale that uses bio-electrical impedance analysis for body composition analysis. Height was measured with a Harpended™ stadiometer, which measures the length of curved line staffage to the nearest 0.5 cm.

Blood (5 ml) samples were collected after an overnight fast at the start and end of the 8 week trial period. The blood was collected into vacutainer tubes, and the serum was separated (via centrifugation) and stored (200 μl aliquots) at -20°C until needed for analyses. The concentrations of total cholesterol, triacylglycerol, HDL-cholesterol, LDL-cholesterol, and glucose were measured using commercial diagnostic kits (cholesterol Infinity, triglyceride Infinity, EZ HDL™ cholesterol, EZ LDL™ cholesterol, Glucose Trinder) from SIGMA Diagnostics. C-reactive protein was measured using an ELISA method (BioCheck™ hsC-Reactive Protein ELISA kit, Foster City, CA USA).

### Statistical analyses

The data for each parameter was summarized via n, mean, and standard deviation for Week 0 and Week 8 and the percent difference (Week 8 – Week 0/week 0). The percent change from baseline was tested for differences using analysis of variance. Contrasts were used for testing pair-wise differences.

## Results

### Anthropomorphic characteristics

Waist circumference is an extremely important determinant in the diagnosis of obesity and metabolic syndrome. As shown in Table 2, the significant reduction in this variable across all treatment groups was paralleled by significant reductions in weight and BMI for the two obese treatment groups.

**Table 2:** Effectiveness of *Cissus quadrangularis *formulation on anthropomorphic characteristics: Percent difference in means from Week 0 to Week 8

BMI	Greater than 30 (Obese)			25 – 29 (Overweight)
Treatment	Placebo (n = 30)	Formulation/no diet (n = 31)	Formulation/Diet (n = 31)	Formulation/no diet (n = 31)

Anthropomorphic Characteristics*				
Weight (%)	-2.4 (4.9)	-6.9 (6.8)^c^	-8.5 (11.3)^a^	-4.8 (6.0)
Fat (%)	-1.9 (9.6)	-6.0 (8.4)	-8.0 (7.6)^b^	-4.7 (9.4)
Body Mass Index (%)	-1.0 (4.2)	-8.3 (3.8)^a^	-9.9 (2.7)^a^	-3.9 (3.0)^a^
Waist Circumference (%)	-2.0 (7.2)	-6.6 (4.9)^a^	-8.4 (6.7)^a^	-11.2 (4.7)^a^

* These data are shown as means with standard deviations in parentheses

^a ^P = <0.001 versus placebo

^b ^P = <0.01 versus placebo

^c ^P = <0.05 versus placebo

To translate the percentage loss over 8 weeks into actual measurements, the mean change in weight (kg) for the 3 obese (BMI >30) groups was 95.6 to 93.3 (placebo); 95.8 to 89.2 (formulation/no diet); and 95.3 to 87.2 (formulation/diet). The mean change for the overweight (BMI 25 – 29) group was 76.3 to 72.6 kg (formulation/no diet).

Thus, over a period of 8 weeks, the placebo group lost 2.3 kg; the overweight group lost 3.7 kg and the two obese groups lost 6.6 kg and 8.1 kg, respectively.

### Serological characteristics

As shown in Table 3, there was a significant improvement in virtually every measurement for the three treatment groups vs. placebo. Eight-week use of the *Cissus *formulation significantly reduced plasma total cholesterol and LDL cholesterol in the three treatment groups and increased HDL cholesterol in the two obese groups by 50.5 % and 43.0% The increase in the concentration of circulating HDL-cholesterol in the three treatment groups shows a large reduction in the ratio of total cholesterol to HDL-cholesterol ratios as well as LDL-cholesterol to HDL-cholesterol ratios. All three treatment groups also demonstrated a significant decrease in triglycerides, C- reactive protein, and fasting blood glucose levels.

**Table 3:** Effectiveness of *Cissus quadrangularis *formulation on serological characteristics: Percent difference in means from Week 0 to Week 8

BMI	Greater than 30 (Obese)			25 – 29 (Overweight)
Treatment	Placebo (n = 30)	Formulation/no diet (n = 31)	Formulation/Diet (n = 31)	Formulation/no diet (n = 31)

Serological Characteristics*				
Total Cholesterol (%)	-3.1 (28.7)	-27.0 (14.6)^a^	-26.0 (13.3)^a^	-18.8 (17.0)^b^
LDL Cholesterol (%)	-10.4 (13.4)	-18.4 (9.8)^c^	-32.4 (15.5)^a^	-26.4 (12.8)^a^
HDL Cholesterol (%)	17.4 (38.8)	50.5 (50.7)^b^	43.0 (39.5)^c^	19.6 (37.0)
Triglycerides (%)	-4.5 (25.4)	-36.8 (17.8)^a^	-28.0 (12.2)^a^	-15.0 (15.3)^c^
C-Reactive Protein (%)	0.8 (13.2)	-20.8 (11.2)^a^	-20.8 (10.0)^a^	-16.3 (13.2)^a^
Fasting Blood Glucose (%)	-4.6 (14.8)	-13.4 (13.6)^b^	-16.1 (13.4)^a^	-11.4 (11.8)^c^

* These data are shown as means with standard deviations in parentheses

^a ^P = <0.001 versus placebo

^b ^P = <0.01 versus placebo

^c ^P = <0.05 versus placebo

To translate the percentage loss over 8 weeks into actual measurements, the mean change in Total cholesterol (mg/dl) for the 3 obese (BMI >30) groups was 160.8 to 155.8 (placebo); 159.1 to 116.1 (formulation/no diet); and 171.0 to 126.5 (formulation/diet. The mean change for the overweight (BMI 25 – 29) group was 152.6 to 123.9.

Thus, over a period of 8 weeks, Total cholesterol declined 5.0 mg/dl for the placebo group; 28.7 mg/dl for the overweight group; and 43.0 mg/dl and 44.5 mg/dl for the two obese groups.

#### Adverse events

Adverse events with an incidence >5 included headache (12), gas (11), dry mouth (7), diarrhea (7), and insomnia (6). Since the incidence of all reported side effects was always higher in the placebo group than in any of the treatment groups, it is probably safe to conclude that the *Cissus formulation *had few, if any, negative side effects.

## Discussion

Our results support the hypothesis that the use of a *Cissus quadrangularis *formulation has efficacy in the management of weight loss and metabolic syndrome, particularly for central obesity. The use of the formulation (which also contains green tea, soy, chromium, selenium, and B-vitamins) over an 8-week period brought about a significant reduction in many of the anthropomorphic measures: weight, % body fat, BMI and, especially, waist circumference of obese and overweight patients, regardless of calorie-controlled diet (see Table 2).

Waist circumference is a particularly important factor in weight and body-fat assessment since fat distribution, rather than total body fat, is currently the key indicator of weight-related health problems. The dramatic reduction in waist circumference that accompanied the 8-week use of the *Cissus quadrangularis *formulation for both the diet and no diet groups is particularly important because waist circumference is a major criterion in the diagnosis of obesity and metabolic syndrome [2] and is generally considered a surrogate measure for abdominal visceral fat [12,13]. Moreover, not only does the risk of type 2 diabetes increase with the degree and duration of obesity it, too, is associated with central obesity [6,7].

The reduction in the primary anthropomorphic measurements can be attributed to the synergistic effects of Cylaris™, *Glycine max *extract, *Camellia sinensis *extract and ChromeMate™ ingredients in the formula, all of which have previously been shown to affect weight loss activity [32,35,39,41,42].

*Cissus quadrangularis *phytosterols and fiber extracts have been shown to have anti-lipase, and anorexiant properties that reduce the absorption of dietary fats and enhance satiation by increasing serum serotonin levels [47]. In 1999, Swiss researchers found that men who were given a combination of caffeine and green tea catechin extract burned more calories than those given only caffeine or a placebo. It was postulated that the catechins and caffeine combination sustained the effect of norepinephrine on thermogenesis longer than caffeine alone [39]. Numerous studies on chromium supplementation have also demonstrated weight loss in overweight and obese people [48,49]. Grant et al. (1997) reported that chromium with a moderate diet and exercise regimen influences weight loss and body composition [50].

The modification of certain serological characteristics (blood parameters) (see Table 3) by *Cissus *formulation may or may not be dependent on weight loss. Only the reduction of LDL-cholesterol followed a similar pattern to weight loss over the 8-week trial period. The circulating concentration of total cholesterol and triglycerides, though reduced by the formulation, could be independent of weight loss since all treatment groups (diet/no diet) showed significant cholesterol reduction. The increase in the concentration of circulating HDL-cholesterol in the treatment groups shows a reduction in the ratio of total cholesterol to HDL-cholesterol as well as LDL-cholesterol to HDL-cholesterol. This reduced ratio also implies a reduction in the risk of atherosclerosis and coronary heart disease [51].

Although the exact mechanisms for the formulation's cholesterol-lowering ability needs further study, the various ingredients might interact in a manner similar to statins, fibrates, probucol, nicotinic acid or cholesterol absorption inhibitors. Green tea catechins have a number of antioxidant activities related to cholesterol regulation. For example, the inhibition of the oxidation of low-density lipoproteins and the antithrombotic activity both aid in lowering total cholesterol/LDL-cholesterol and increasing HDL-cholesterol levels [52,53]. Also, the phytochemical constituents (phytosterols, β-sitosterol, δ-amyrin and δ-amyrone), in *Cissus quadrangularis *may have activity similar to other plant sterols. The molecular structure of phytosterols, for example, is practically identical to that of cholesterol.

Recent studies show that metabolic syndrome causes an inflammatory process in the blood vessels that leads to arteriosclerosis. This inflammatory process can be gauged by blood levels of C-reactive protein [54] and, as our results showed, *Cissus *formulation significantly reduced the circulating concentrations of CRP thereby inhibiting the inflammatory process and possibly reducing individual components of metabolic syndrome [54]. In metabolic syndrome, the body becomes resistant to insulin, and high levels of glucose remain trapped in the blood. In response, the pancreas produces more insulin. The extra insulin temporarily allows glucose to enter the cells and also increases cholesterol and triglyceride levels.

In sum, *Cissus *formulation administered twice daily to obese and overweight persons with symptoms of metabolic syndrome results in both weight reduction and an improvement in the symptoms associated with metabolic syndrome. It has also shown efficacy in the control and lowering of triglyceride concentrations, total cholesterol, LDL-cholesterol, and fasting blood glucose. The formulation may also have applications in other metabolic diseases, such as diabetes mellitus.

## Authors' contributions

JO conceived, designed, coordinated and drafted the manuscript; DK carried out blood sampling and performed statistical analyses; GA participated in the design and carried out analytical work; CM participated in the design and carried out analytical work; XT participated in the formulation of the extract.

All the authors read and approved the final manuscript.

## References

[B1] Eckel RH, Grundy SM, Zimmet PZ (2005). The metabolic syndrome. Lancet.

[B2] Grundy SM, Brewer HB, Cleeman JI, Smith SC, Lenfant C (2004). Definition of metabolic syndrome: Report of the National Heart, Lung, and Blood Institute/American Heart Association conference on scientific issues related to definition. Circulation.

[B3] Mokdad AH, Ford ES, Bowman BA, Dietz WH, Vinicor F, Bales VS, Marks JS (2003). Prevalence of obesity, diabetes, and obesity-related health risk factors, 2001. JAMA.

[B4] Ford ES, Giles WH, Dietz WH (2002). Prevalence of the metabolic syndrome among US adults: findings from the Third National Health and Examination Survey. JAMA.

[B5] Park YW, Zhu S, Palaniappan L, Heshka S, Carnethon M, Heymsfield S (2003). The metabolic syndrome prevalence and associated risk factor findings in the US population from the Third National Health and Nutrition Examination Survey, 1988–1994. Arch Intern Med.

[B6] Abbasi F, Brown BW, Lamendola C, McLaughlin T, Reaven GM (2002). Relationship between obesity, insulin resistance, and coronary heart disease risk. J Am Coll Cardiol.

[B7] Bogardus C, Lillioja S, Mott DM, Hollenbeck C, Reaven G (1985). Relationship between degree of obesity and in vivo insulin action in man. Am J Physiol.

[B8] Poirier P, Giles TD, Bray GA, Yuling H, Stern J, Pi-Sunyer FX, Eckel RH (2006). Obesity and cardiovascular disease: pathophysiology, evaluation, and effect of weight loss: an update of the 1997 American Heart Association Scientific Statement on Obesity and Heart Disease from the Obesity Committee of the Council on Nutrition, Physical Activity, and Metabolism. Circulation.

[B9] Wilson PW, D'Agostino RB, Sullivan L, Parise H, Kannel WB (2002). Overweight and obesity as determinants of cardiovascular risk: the Framingham experience. Arch Intern Med.

[B10] Denke MA, Sempos CT, Grundy SM (1994). Excess body weight: an under-recognized contributor to high blood cholesterol levels in white American women. Arch Intern Med.

[B11] Ochs-Balcom HM, Grant BJ, Muti P, Sempos CT, Freudenheim JL, Trevisan M, Cassano PA, Iacoviello L, Schunemann HJ (2006). Pulmonary function and abdominal adiposity in the general population. Chest.

[B12] Paek KW, Hong YM (2006). Health behavior factors affecting waist circumference as an indicator of abdominal obesity. J Prev Med Pub Health.

[B13] Kuk JL, Katzmarzyk PT, Nichaman MZ, Church TS, Blair SN, Ross R (2006). Visceral fat is an independent predictor of all-cause mortality in men. Obesity.

[B14] Pouliot MC, Després JP, Lemieux S, Moorjani S, Bouchard C, Tremblay A, Nadeau A, Lupien PJ (1994). Waist circumference and abdominal sagittal diameter: best simple anthropometric indexes of abdominal adipose tissue accumulation and related cardiovascular risk in men and women. Am J Cardiol.

[B15] Knowler WC, Barrett-Connor E, Fowler SE, Hamman RF, Lachin JM, Walker EA, Nathan DM (2002). Reduction in the incidence of type 2 diabetes with lifestyle intervention or metformin. N Engl J Med.

[B16] Pyorala K, Ballantyne CM, Gumbiner B, Lee MW, Shah A, Davies MJ, Mitchel YB, Pedersen TR, Kjekshus J (2004). Reduction of cardiovascular events by Simvastatin in nondiabetic coronary heart disease patients with and without the metabolic syndrome: Subgroup analyses of the Scandinavian Simvastatin Survival Study (4S). Diabetes Care.

[B17] Buchanan TA, Xianag AH, Peters RK, Kjos SL, Marroquin A, Goico J, Ochoa C, Tan S, Berkowitz K, Hodis HN, Azen SP (2002). Preservation of pancreatic β-cell function and prevention of type 2 diabetes by pharmacological treatment of insulin resistance in high risk Hispanic women. Diabetes.

[B18] Chanoine JP, Hampl S, Jensen C, Boldrin M, Hauptman J (2002). Effect of orlistat on weight and body composition in obese adolescents: a randomized controlled trial. JAMA.

[B19] Lacey LA, Wolf A, O'shea D, Erny S, Ruof J (2005). Cost-effectiveness of orlistat for the treatment of overweight and obese patients in Ireland. Int J Obes.

[B20] Swinburn BA, Carey D, Hills AP, Hooper M, Marks S, Proietto J, Strauss BJ, Sullivan D, Welborn TA, Caterson ID (2005). Effect of orlistat on cardiovascular disease risk in obese adults. Diabetes Obes Metab.

[B21] Melia AT, Koss-Twardy SG, Zhi J (1996). The effect of orlistat, an inhibitor of dietary fat absorption, on the absorption of vitamins A and E in healthy volunteers. J Clin Pharmacol.

[B22] Centers for Disease Control and Prevention (CDC) (1997). Cardiac valvulopathy associated with exposure to fenfluramine or dexfenfluramine: U.S. Department of Health and Human Services interim public health recommendations, November 1997. MMWR Morb Mortal Wkly Rep.

[B23] Chopra SS, Patel MR, Gupta LP, Datta IC (1975). Studies on Cissus quadrangularis in experimental fracture repair: effect on chemical parameters in blood. Indian J Med Res.

[B24] Deka DK, Lahon LC, Saikia J, Mukit A (1994). Effect of *Cissus quadrangularis *in accelerating healing process of experimentally fractured radius-ulna of dog, a preliminary study. Indian J of Pharmacol.

[B25] Udupa KN, Prasad GC (1964). Further studies on the effect of Cissus quadrangularis in accelerating fracture healing. Indian J Med Res.

[B26] Udupa KN, Prasad GC (1964). Biomechanical and calcium 45 studies on the effect of Cissus quadrangularis in fracture repair. Indian J Med Res.

[B27] Udupa KN, Prasad GC (1963). Effect of Cissus quadrangularis on the healing of cortisone treated fractures. Indian J Med Res.

[B28] Udupa KN, Prasad GC (1962). Cissus quadrangularis in healing of fractures. A clinical study. J Indian Med Assoc.

[B29] Chidambara Murthy KN, Vanitha A, Mahadeva Swamy M, Ravishankar GA (2003). Antioxidant and antimicrobial activity of cissus quadrangularis L. J Med Food.

[B30] Das PK, Sanyal AK (1964). Studies on Cissus quadrangularis linn, acetylcholine like action of the total extract. Indian J Med Res.

[B31] Singh SP, Mishra N, Dixit KS, Singh N, Kohli RP (1984). An experimentally study of analgesic activity of Cissus quadrangularis. Indian J of Pharmacol.

[B32] Oliver-Bever B (1983). Medicinal plants in tropical West Africa. II. Plants acting on the nervous system. J Ethnopharmacol.

[B33] Oliver-Bever B (1983). Medicinal plants in tropical West Africa. III. Anti-infection therapy with higher plants. J Ethnopharmacol.

[B34] Ramaamurthy B, Seshadri T (1939). Carotene content of *Vitis quadrangularis*. Proceedings of the Indian Academy of Sciences.

[B35] Sen SP (1964). Study of the active constituents (ketosteroids) of *Cissus quadrangularis*. Indian J of Pharmacol.

[B36] Mehta M, Kaur N, Bhutani KK (2001). Determination of marker constituents from *Cissus quadrangularis *Linn. and their quantitation by HPTLC and HPLC. Phytochem Anal.

[B37] Shirwaikar A, Khan S, Malini S (2003). Antiosteoporotic effect of ethanol extract of Cissus quadrangularis Linn. on ovariectomized rat. J Ethnopharmacol.

[B38] Combaret L, Taillandier D, Dardevet D, Bechet D, Ralliere c, Claustre A, Grizard J, attaix D (2004). Glucocorticoids regulate mRNA levels for subunits of the 19 S regulatory complex of the 26 S proteasome in fast-twitch skeletal muscles. Biochem J.

[B39] Dulloo AG, Duret C, Rohrer D, Girardier L, mensi N (1999). Efficacy of a green tea extract rich in catechin polyphenols and caffeine in increasing 24-h energy expenditure and fat oxidation in humans. Am J Clin Nutr.

[B40] Dulloo AG, Seydoux J, Girardier L, Chantre P, vandermander J (2000). Green tea and thermogenesis: interactions between catechin-polyphenols, caffeine and sympathetic activity. Int J Obes Relat Metab Disord.

[B41] Anderson JW, Johnstone BM, Cook-Newell ME (1995). Meta-analysis of the effects of soy protein intake on serum lipids. N Engl J Med.

[B42] Anderson RA (1997). Chromium as an essential nutrient for humans. Regul Toxicol Pharmacol.

[B43] Yang X, Li SY, Dong F, Ren J, Sreejayan N (2006). Insulin-sensitizing and cholesterol-lowering effects of chromium (d-Phenylalanine)(3). J Inorg Biochem.

[B44] Rayman MP (2000). The importance of selenium to human health. Lancet.

[B45] Manore MM (2000). Effect of physical activity on thiamine, riboflavin, and vitamin B-6 requirements. Am J Clin Nutr.

[B46] Seshadri N, Robinson K (2000). Homocysteine, B vitamins, and coronary artery disease. Med Clin North Am.

[B47] Oben J, Mandob D, Fomekong G, Agbor G, Momo C The effect of an extract of *Cissus quadrangularis *on weight and serum lipids in obese patients in Cameroon: a randomized double blind clinical trial.

[B48] Crawford V, Scheckenbach R, Preuss HG (1999). Effects of niacin-bound chromium supplementation on body composition in overweight African-American women. Diabetes Obes Metab.

[B49] Lefavi RG, Anderson RA, Keith RE, Wilson GD, McMillan JL, Stone MH (1992). Efficacy of chromium supplementation in athletes: emphasis on anabolism. Int J Sport Nutr.

[B50] Grant KE, Chandler RM, Castle AL, Ivy JL (1997). Chromium and exercise training: effect on obese women. Med Sci Sports Exerc.

[B51] Kannel WB (2000). Incidence and epidemiology of heart failure. Heart Fail Rev.

[B52] Kang WS, Lim IH, Yuk DY, Chung KH, Park JB, Yoo HS, Yun YP (1999). Antithrombotic activities of green tea catechins and (-)-epigallocatechin gallate. Throm Res.

[B53] Miura Y, Chiba T, Miura S, Tomita I, Umegaki K, Ikeda M, Tomita T (2000). Green tea polyphenols (flavan 3-ols) prevent oxidative modification of low density lipoproteins: an ex vivo study in humans. J Nutr Biochem.

[B54] Haffner SM (2006). The metabolic syndrome: inflammation, diabetes mellitus, and cardiovascular disease. Am J Cardiol.

[B55] Nash DT (2005). Relationship of C-reactive protein, metabolic syndrome and diabetes mellitus: potential role of statins. J Natl Med Assoc.

